# Chemical Modification of Curcumin into Its Semi-Synthetic Analogs Bearing Pyrimidinone Moiety as Anticancer Agents

**DOI:** 10.3390/plants11202737

**Published:** 2022-10-16

**Authors:** Obaid Afzal, Mohammad Yusuf, Mohamed Jawed Ahsan, Abdulmalik S. A. Altamimi, Md. Afroz Bakht, Amena Ali, Salahuddin

**Affiliations:** 1 Department of Pharmaceutical Chemistry, College of Pharmacy, Prince Sattam Bin Abdulaziz University, Al-Kharj 11942, Saudi Arabia; 2 Department of Clinical Pharmacy, College of Pharmacy, Taif University, P.O. Box 11099, Taif 21944, Saudi Arabia; 3 Department of Pharmaceutical Chemistry, Maharishi Arvind College of Pharmacy, Jaipur 302 039, India; 4 Department of Chemistry, College of Science and Humanity Studies, Prince Sattam Bin Abdulaziz University, P.O. Box 83, Al-Kharj 11942, Saudi Arabia; 5 Department of Pharmaceutical Chemistry, College of Pharmacy, Taif University, P.O. Box 11099, Taif 21944, Saudi Arabia; 6 Department of Pharmaceutical Chemistry, Noida Institute of Engineering and Technology (Pharmacy Institute), Knowledge Park-2, Greater Noida 201306, India

**Keywords:** antiproliferative activity, anti-EGFR, curcumin analogues, pyrimidinone, synthesis, molecular docking

## Abstract

Natural products (NPs) continue to provide a structural template for the design of novel therapeutic agents and expedite the drug discovery process. The majority of FDA-approved pharmaceuticals used in medical practice can be traced back to natural sources, and NPs play a significant role in drug development. Curcumin, one of the most well-studied chemicals among the NPs, is currently the subject of intense investigation for its biological effects, including the prevention and treatment of cancer. Cancer has overtaken all other causes of death in the world today, with 19.3 million new cases and nearly 10 million deaths predicted in 2020. In the present investigation, we reported the synthesis of three semi-synthetic analogues of curcumin-bearing pyrimidinone moiety by the chemical modification of the diketone function of curcumin followed by their characterization by analytical techniques including infrared (IR), nuclear magnetic resonance (NMR), and mass spectral data. According to the National Cancer Institute (NCI US) methodology, the curcumin analogues (**C1-C3**) were tested for their anticancer efficacy against 59 cancer cell lines in a single dose assay. 1-(2,6-Dichlorophenyl)-4,6-bis((E)-4-hydroxy-3-methoxystyryl)pyrimidin-2(1*H*)-one (**C2**) demonstrated the most promising anticancer activity with mean percent growth inhibition (%GIs) of 68.22 in single dose assay at 10 µM. The compound exhibited >68 %GIs against 31 out of 59 cancer cell lines and was found to be highly active against all leukemia and breast cancer cell lines. The compound **C2** showed a lethal effect on HT29 (colon cancer) with %GI of 130.44, while 99.44 %GI was observed against RPMI-8226 (Leukemia). The compound **C2** displayed better anticancer activity against the panels of CNS, melanoma, ovarian, prostate, and breast cancer cell lines than curcumin and other anti-EGFR agents gefitinib and imatinib in single dose assay. The compound **C2** also demonstrated potent anticancer activity in a 5-dose assay (0.001 to 100 µM) with GI_50_ values ranging from 1.31 to 4.68 µM; however, it was found to be non-selective with SR values ranging from 0.73 to 1.35. The GI_50_ values of compound **C2** were found to be better than that of the curcumin against all nine panels of cancer cell lines. All of the curcumin analogues were subsequently investigated for molecular docking simulation against EGFR, one of the most attractive targets for antiproliferative action. In molecular docking studies, all the ligands were found to accommodate the active site of EGFR and the binding affinity of ligand **C2** was found to be −5.086 kcal/mol. The ligand **C2** exhibited three different types of interactions: H-bond (Thr790 and Thr854), π-cationic (Arg841), and aromatic H-bond (Asn842). The curcumin analogues reported in the current investigation may provide valuable therapeutic intervention for the prevention and treatment of cancer and accelerate anticancer drug discovery programs in the future.

## 1. Introduction

**Figure 1:**
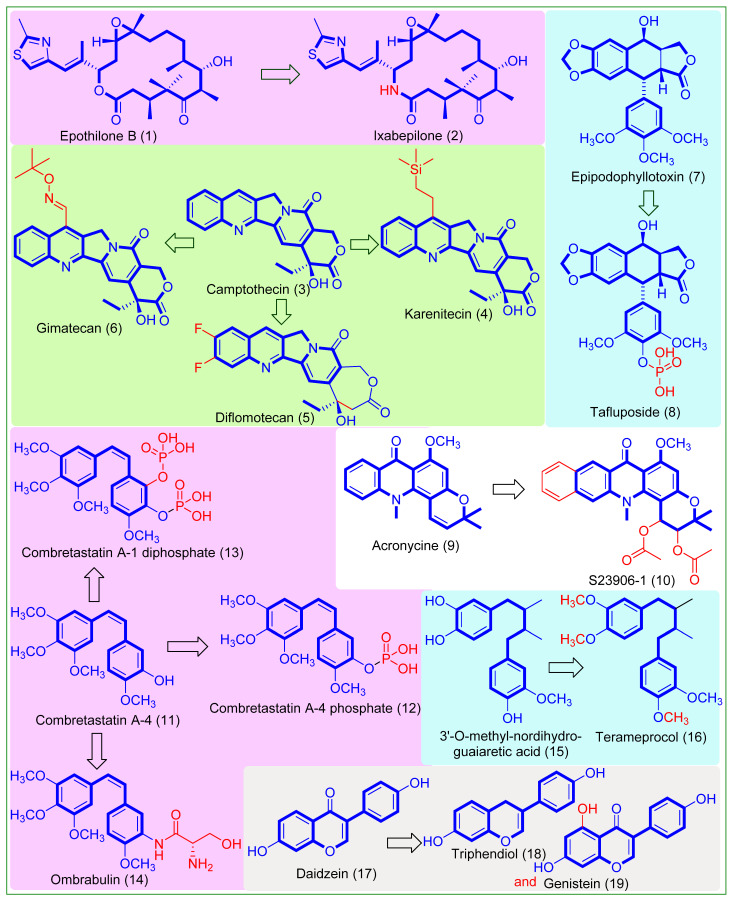
Anticancer drugs derived from natural products [NPs; epothilone B (**1**), camptothecin (**3**), epipodophyllotoxin (**7**), acronycine (**9**), combretastatin A-4 (**11**), 3’-*O*-methylnordihydroguaiaretic acid (**15**), and daidzein (**17**)] by chemical modification.

**Figure 2:**
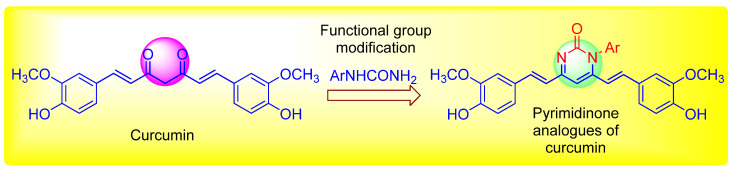
Structural modification of diketone function into pyrimidinone.

Currently, a major challenge facing the modern scientific community is the development of novel anticancer drugs with fewer side effects. Research on natural products (NPs) has been boosted recently because NPs are thought to be relatively safer than synthetic ones [1,2]. The use of NPs for medicinal purposes has gained popularity during the past few decades. Many of the FDA-approved medications used in clinical practices came from natural sources [3,4]. Many anticancer agents have been obtained by the chemical modification of NPs. A few examples of such modifications are given in Figure 1 [5,6,7,8,9,10,11,12,13,14]. Similarly, curcumin, one of the key chemical components found in turmeric (*Curcuma longa Linn.*), has been utilized to prepare a variety of semi-synthetic analogues [15,16,17,18,19,20,21,22]. Medicinal chemists have identified four main sites to bring about chemical modification in curcumin to form semi-synthetic congeners, including active methylene (-CH_2_-), aryl side chain, diketone group, and carbon-carbon double bonds (-CH=CH-) with improved bioactivity [23,24]. In the current study, we described the chemical modification of the diketone function of curcumin into their pyrimidine analogues as well as their antiproliferative activity. The chemical modification is outlined in Figure 2. The structural alteration was found to enhance biological activities by enhancing stability, reducing rotational freedom, and diminishing metal-chelation characteristics [25]. Our research team found that curcumin analogues have been demonstrated to have anticancer, antimalarial, and anti-HIV effects in the past [26,27,28]. Numerous biological activities, such as those that are antibacterial, anticancer, antioxidant, antimalarial, anti-inflammatory, anti-Alzheimer’s, and anti-HIV, have been reported [15,16,17,18,19,20].

Cancer has overtaken all other causes of death in the world today, with 19.3 million new cases and nearly 10 million deaths predicted in 2020 [29]. Nowadays, systemic chemotherapy is used in conjunction with large-mass surgical excision of the tumor and radiation therapy. Chemotherapy often associated with numerous toxic effects and scientists from all over the world are working to find safer cancer treatments. As active ingredients derived from natural sources were presumed to be safe, our reliance on nature increased. The majority of anticancer drugs today in clinical practices are from natural sources [4,30]. The epidermal growth factor receptor (EGFR) is found on the surface of some normal cells and is involved in cell growth. The EGFR protein participates in cell signaling pathways that regulate cell survival and division. Sometimes, mutations in the EGFR gene cause EGFR proteins to be made in higher than normal amounts in a number of cancer cell lines, including those from breast, colon, non-small cell lung, renal, melanoma, ovarian, and prostate cancers. This causes cancer cells to divide more rapidly [31,32,33,34,35]. Curcumin analogues have also been identified as EGFR inhibitors, hence we selected EGFR as a potential target for molecular docking studies [2,22,26]. We examine the binding insight of curcumin analogues against the active site since it is a rational target for several anticancer treatments (such as Gefitinib, Erlotinib, Cetuximab, Panitumumab, and others) as well as the most widely researched receptor in the tyrosine kinase superfamily [36,37,38,39].

## 2. Results

### 2.1. Isolation of Curcumin

Curcumin was isolated by a conventional method as per the reported procedure and nearly 250 mg of curcumin was isolated with 80 g of the ground tumeric powder [2,40].

### 2.2. Chemistry

#### Preparation of 1-aryl-4,6-bis((E)-4-hydroxy-3-methoxystyryl)pyrimidin-2(1H)-one (C1-C3)

**Scheme 1:**
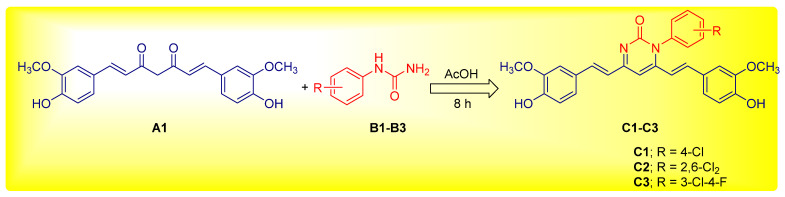
Protocol for the synthesis of semi-synthetic curcumin analogues (**C1-C3**).

**Scheme 2:**
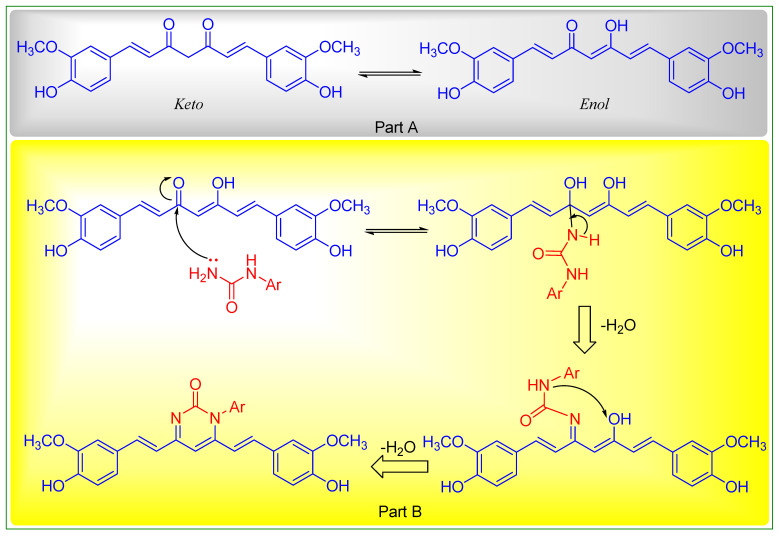
The reaction mechanism for the synthesis of curcumin analogues (**C1-C3**).

The curcumin analogues (**C1-C3**) bearing pyrimidinone moiety were synthesized by conventional heating method and the synthetic protocol is summarized in Scheme 1. A mixture of isolated curcumin (**A1**) (0.20 mmol; 73.6 mg) and substituted phenyl urea (**B1-B3**) (0.20 mmol) in glacial acetic acid (10 mL) was stirred in a sandbath at 80 °C for 8 h. The reaction mixture was then concentrated and poured into the crushed ice to obtain crude final products (**C1-C3**). The crude product was then re-crystallized with ethanol to obtain 1-substitutedphenyl-4,6-bis((E)-4-hydroxy-3-methoxystyryl)pyrimidin-2(1H)-one (**C1-C3**). The completion of the reaction was monitored throughout by preparatory thin layer chromatography (TLC Silica gel 60 F_254_) in the mobile phase n-hexane: ethylacetate (6: 4). The substituted phenyl urea ((**B1-B3**) was prepared as per the reported method [41,42]. Curcumin exhibits tautomeric isomerism viz. keto and enol form (Part A). The formation of curcumin is supposed to be accomplished in two steps as shown in Scheme 2 (Part B).

### 2.3. Antiproliferative Activity

**Figure 3:**
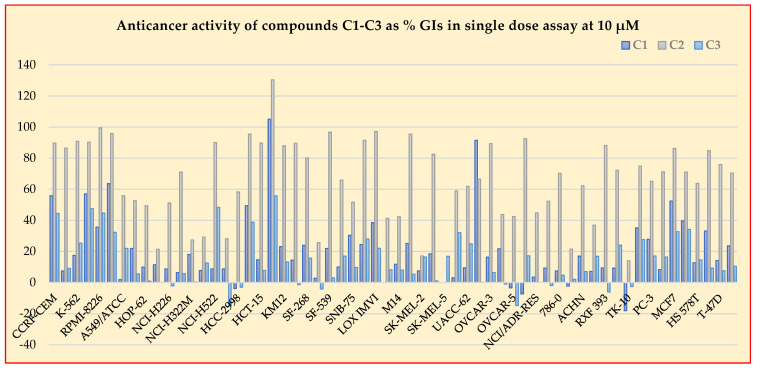
The percent growth inhibition of compounds **C1-C3** in single dose assay against 59 NCI cancer cell lines.

The anticancer activity of the curcumin analogues (**C1-C3**) was carried out against 60 NCI cancer cell lines derived from nine different panels (breast, colon, CNS, leukemia, melanoma, non-small cell lung, ovarian, renal, and prostate cancer cell lines) at a single dose (10 µM) and five dose assay as per the National Cancer Institute US [43,44,45,46]. The results of anticancer activity of compounds **C1-C3** in single dose assay at 10 µM are given in the Table 1 (Figures S1–S3). The compound **C1** displayed more than 50 to 67 percent growth inhibitions (% GIs) against three leukemia cancer cell lines (SR, MOLT-4, and CCRF-CEM) with %GIs of 63.58, 57.03, and 55.82 respectively, and one breast cancer cell line MCF7 (%GIs of 52.38). The compound **C1** showed moderate anticancer activity with %GI of 20–50 against 16 cancer cell lines HCT116 (%GI = 49.42), MDA-MB-231 (%GI = 39.69), LOX IMVI (%GI = 38.43), RPMI-8226 (%GI = 35.53), UO-31 (%GI = 35.12), BT-549 (%GI = 33.10), SNB-75 (%GI = 30.28), PC-3 (%GI = 27.72), MDA-MB-435 (%GI = 25.07), U251 (%GI = 24.47), SF-268 (%GI = 23.95), MDA-MB-468 (%GI = 23.56), KM12 (%GI = 23.01), SF-539 (%GI = 21.92), EKVX (%GI = 21.89), and OVCAR-4 (%GI = 21.66), while showed less activity against rest of the 37 cancer cell lines. The compound **C1** displayed the most promising anticancer activity against IGROV1 with %GI of 91.43. The compound **C1** displayed significant anticancer activity against the colon cancer cell line HT29 with %GI of 55.65, while displayed 40–50 %GIs against four cancer cell lines viz. NCI-H522 (%GI = 48.31), MOLT-4 (%GI = 47.47), RPMI-8226 (%GI = 44.65), and CCRF-CEM (%GI = 44.43). The compound **C3** displayed 20–40 %GIs against 12 cancer cell lines, HCT-116 (%GI = 38.73), MDA-MB-231 (%GI = 34.16), MCF7 (%GI = 32.66), SR (%GI = 32.20), UACC-257 (%GI = 32.02), U251 (%GI = 27.95), UO-31 (%GI = 27.54), K-562 (%GI = 25.32), UACC-62 (%GI = 24.86), SN-12C (%GI = 23.96), LOX IMVI (%GI = 21.97), and A529 (%GI = 21.87), while it displayed less activity against the rest of the 42 cancer cell lines. The compounds **C1** and **C2** displayed lethal effects on the colon cancer cell line HT29 with %GI of 105.09 and 130.44 respectively. The compound **C2** displayed the most promising anticancer activity among the series. The compound **C2** displayed 50–67 %GIs against 11 cancer cell lines IGROV1 (%GI = 66.54), SNB-19 (%GI = 65.86), PC-3 (%GI = 65.14), HS-578T (%GI = 63.69), ACHN (%GI = 62.22), UACC-62 (%GI = 61.83), UACC-257 (%GI = 58.9), HCC-2998 (%GI = 58.23), A549 (%GI = 55.85), EKVX (%GI = 52.61), SK-OV-3 (%GI = 52.29), SNB-75 (%GI = 51.58), and NCI-H226 (%GI = 51.15), and displayed 20–50 %GIs against HOP-62 (%GI = 49.42), NCI/ADR-RES (%GI = 44.79), OVCAR-4 (%GI = 43.74), OVCAR-5 (%GI = 42.42), M14 (%GI = 42.29), MALME-3M (%GI = 41.21), CAKI-1 (%GI = 36.82), COLO 205 (%GI = 29.19), NCI-H460 (%GI = 29.16), NCI-H322M (%GI = 27.35), SF-295 (%GI = 25.63), A498 (%GI = 21.46), and HOP-92 (%GI = 21.31), while displaying less activity with %GIs <20 against 2 cancer cell lines namely SK-MEL-2 (%GI = 17.08) and TK-10 (%GI = 13.98). The compound **C2** displayed the most promising anticancer activity with %GIs more >68 against 30 cell lines namely RPMI-8226 (%GI = 99.44), LOX IMVI (%GI = 97.17), SF-539 (%GI = 96.65), SR (%GI = 95.88), MDA-MB-435 (%GI = 95.43), HCT-116 (%GI = 95.37), OVCAR-8 (%GI = 92.47), U251 (%GI = 91.46), K-562 (%GI = 90.79), MOLT-4 (%GI = 90.29), NCI-H522 (%GI = 90.11), HCT-15 (%GI = 89.72), CCRF-CEM (%GI = 89.66), SW-620 (%GI = 89.57), OVCAR-3 (%GI = 89.34), RXF 393 (%GI = 88.18), KM12 (%GI = 87.88), MCF7 (%GI = 86.25), BT-549 (%GI = 84.83), SK-MEL-28 (%GI = 82.41), SF-268 (%GI = 80.15), T-47D (%GI = 75.89), UO-31 (%GI = 74.86), SN 12C (%GI = 72.16), DU-145 (%GI = 71.26), NCI-H23 (%GI = 71.04), MDA-MB-231 (%GI = 71.00), MDA-MB-468 (%GI = 70.41), 786-O (%GI = 70.30), and HL-60(TB) (%GI = 46.47). The anticancer activity of curcumin analogues (**C1-C3**) is shown in Figure 3. The anticancer activity of curcumin analogues **C1-C3** were compared with curcumin and other EGFR inhibitors, Gefitinib and Imatinib, and their comparative anticancer activity at 10 µM is given in Table 2. The mean %GIs of individual panels was calculated from the single dose assay data. The compound **C2** displayed better anticancer than curcumin and standard drug imatinib and gefitinib activity against CNS, melanoma, ovarian, prostate, and breast cancer cell lines. The curcumin analogues displayed mean growth percent inhibition of 68.22 (%GI = >68), and were further selected for screening in a 5-dose assay [47].

**Figure 4:**
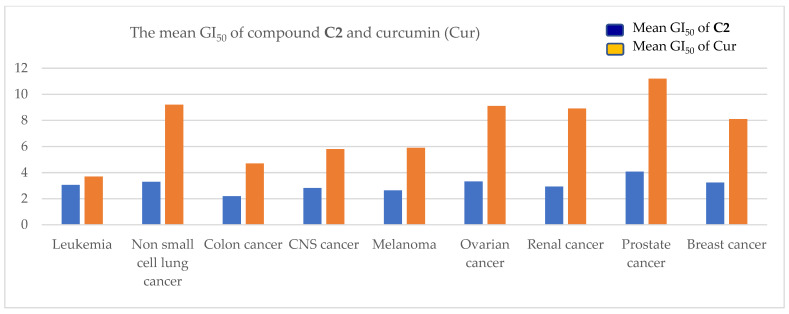
The mean GI_50_ for compound **C2** and curcumin (Cur) in µM concentration.

**Figure 5:**
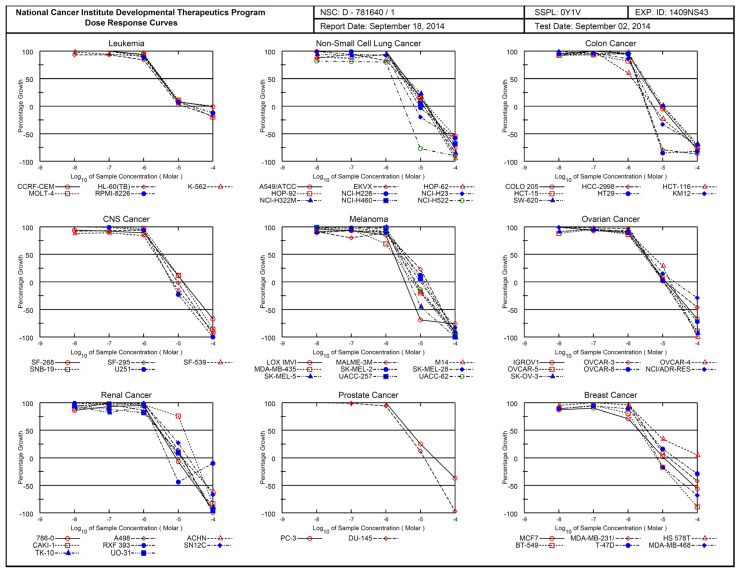
58 NCI cancer cell lines based anticancer activity of compound **C2**.

The 5-dose assay was carried out as per the reported method [48,49,50]. The compound **C2** showed strong antiproliferative activity in a 5-dose assay against 58 NCI cell lines, with GI_50_ values ranging from 1.31 to 4.68 µM, TGI values ranging from 1.05 to >100 µM, and LC_50_ values between 6.41 and >100 µM. The compound **C2** displayed superior anticancer activity than curcumin in 5-dose assay (Figure 4). The compound **C2** displayed the most promising antiproliferative activity against HL-60(TB) (GI_50_ = 2.64 µM) among leukemia cell lines, NCI-H522 (GI_50_ = 1.55 µM) among non-small cell lung cancer cell lines, HCT-116 (GI_50_ = 1.31 µM) among colon cancer cell lines, SF-539 (GI_50_ = 2.18 µM) among CNS cancer cell lines, MDA-MB-435 (GI_50_ = 1.63 µM) among melanoma cell lines, OVCAR-5 (GI_50_ = 2.76 µM) among ovarian cancer cell lines, CAKI-1 (GI_50_ = 1.46 µM) among renal cell lines, DU-145 (GI_50_ = 3.68 µM) among prostate cancer cell lines, and MCF7 (GI_50_ = 2.00 µM) among breast cancer cell lines. The compound **C2** exhibited non-selectivity against all the nine panels of cancer cell lines with a selectivity ratio (SR) ranging between 0.73 and 1.35 as the value of SR was found to be less than three (Table 1) [51]. The anticancer activity of compounds **C2** against nine panels of 58 NCI cancer cell lines in terms of GP and Log_10_ molar concentration are shown in Figure 5.

### 2.4. Molecular Docking Studies

**Figure 6:**
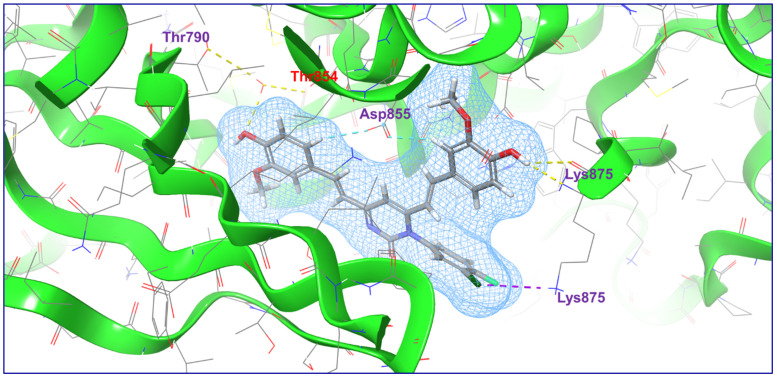
The 3D interactions of ligand **C3** against the active site of EGFR (binding affinity = −4.396 kcal/mol).

**Figure 7:**
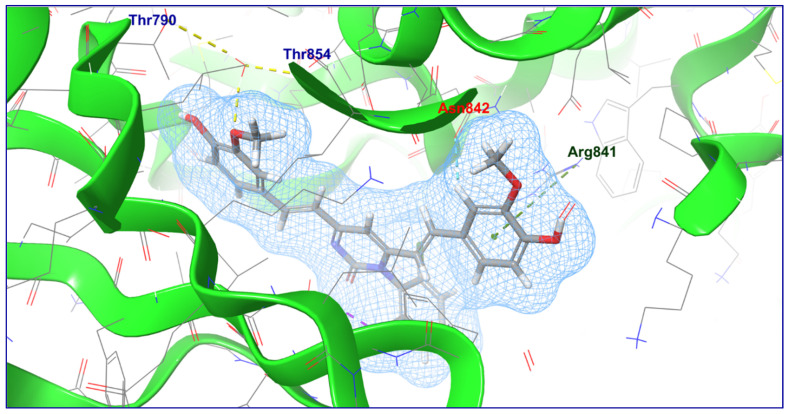
The 3D interactions of ligand **C2** against the active site of EGFR (binding affinity = −5.086 kcal/mol).

Curcumin and its analogues’ anti-EGFR action were well-documented in the literature [2,22,26]. The molecular docking against EGFR (PDB ID: 2J5F) was carried out at the binding site of 34-JAB in the current work as per the reported protocol [52]. The molecular docking score and types of interaction of curcumin analogues are summarized in Table 3. Three types of interaction were observed for the ligands (**C1–C3**) including H-bond, π-cationic, and halogen bonds with a binding affinity of −4.936 to −5.117 kcal/mol. The ligand **C1** showed an aromatic H-bond with the residue Asp855 (Figure S4). The ligand **C3** showed two types of interactions including H-Bond (with residue Thr790, Thr854, Lys875 via water molecule); halogen bond (with the residue Lys875); aromatic H-bond (with the residue Asp855) (Figure 6). The ligand **C2** was found to be the most promising compound and displayed the most significant anticancer activity. The ligand **C2** showed three types of interactions including H-bond of methoxy function of phenyl ring with the residue Thr854 via water molecule, π-cationic interaction of the 4-hydroxy-3-methoxyphenyl with the residue Arg841, and halogen bond interaction of one of the *o*-chloro function with the water molecule. Furthermore, the ligand **C2** displayed good interaction with residues Ala743, Val726, Thr790, Lys745, Leu799, Asp800, Gly721, Leu781, Cys775, Leu844, Cys797, Ser720, and Phe723. The 3D interactions of ligand **C2** against the active site of EGFR are shown in Figure 7.

## 3. Discussion

Three new curcumin analogues (**C1-C3**) were reported in the current work. Curcumin and substituted phenyl urea in glacial acetic acid were stirred at 80 °C for 8 h to obtain 1-aryl-4,6-bis((E)-4-hydroxy-3-methoxystyryl)pyrimidin-2(1H)-ones (**C1-C3**) in good yields (75–80%). All the title compounds were characterized by analytical techniques, followed by their anticancer evaluation and molecular docking studies. One of the curcumin analogues (**C2**) displayed the most promising anticancer activity with a mean GP of 31.78 (% GI = 68.22). Nearly 31 cancer cell lines were found to be highly sensitive against the compound **C2** with %GIs of >68. Two of the curcumin analogues (**C1** and **C2**) displayed lethal effects on HT29 colon cancer cell lines with %GIs of 105.09 and 130.44. The anticancer activity of compound **C2** was found to be better than that of curcumin and anti-EGFR drugs (gefitinib and imatinib) against CNS, melanoma, ovarian, prostate, and breast cancer cell lines, it was also found as active against all the cell lines of leukemia and breast cancer cell line panels (Table 2). The compound **C2** was further studied in 5-dose assay in which GI_50_ values were found to be ranging from 1.31 to 4.68 µM, however, it was found to be non-selective with SR values ranging from 0.73 to 1.35. The GI_50_ values of compound **C2** were found to be better than that of the curcumin against all nine panels of cancer cell lines. The anticancer activity of compound **C2** (mean % GI = 68.22) was found to be promising more than the previously reported work [2]. Chemically altering the diketone function to produce pyrazole and primidone analogues was shown to be more promising than doing so to produce bigenelli type curcumin analogues [2,26,53,54]. Curcumin analogues with 3-chloro-4-fluoro (**C3**) substitution in the phenyl ring at 1-pyrimidine showed mean %GI of 14.33, while 4-chloro (**C1**) substitution showed mean %GI of 20.37 and 2,6-dichloro (**C2**) substitution showed the most promising anticancer activity with %GI of 68.22. Three types of interaction were observed for the ligands (**C1-C3**) including H-bond, π-cationic, and halogen bonds with a binding affinity of -4.936 to -5.117 kcal/mol. The ligands **C1** and **C2** bind to the EGFR active site efficiently, whereas the ligand C3 binds less efficiently. The most active compound (**C2**) showed H-bond interaction with residue Thr854 through the water molecule, π-cationic interaction with residue Arg841, and halogen bond interaction with the water molecule. It also showed good interaction with residues Ala743, Val726, Thr790, Lys745, Leu799, Asp800, Gly721, Leu781, Cys775, Leu844, Cys797, Ser720, and Phe723.

## 4. Materials and Methods

### 4.1. Preparation of Curcumin Analogue C1-C3

An equimolar mixture of curcumin (**A1**) (0.20 mmol; 73.6 mg) and substituted phenyl ureas (**B1-B3**) (0.20 mmol) in glacial acetic acid (10 mL) was stirred in a sand bath at 80 °C for 8 h. After completion, the reaction mixture was concentrated under a vacuum to remove excess solvent and poured into crushed ice, filtered, dried, and recrystallized with ethanol to yield compound **C1-C3 [2,26]**.

#### 4.1.1. 1-(4-Chlorophenyl)-4,6-bis((E)-4-hydroxy-3-methoxystyryl)pyrimidin-2(1H)-one

(**C1**): Mp 132–134 °C; IR (KBr) v_max_: 3382 (OH), 1637 (C=O), 1558 (C=N), 1163 (O-CH_3_), 713 (C-Cl) cm^−1^; ^1^H NMR (400 MHz; DMSO-d_6_): δ ^1^H NMR (400 MHz; DMSO-d_6_): δ; 3.81 (6H, s, OCH_3_), 6.04 (1H, s, ArH_pyrimidine_), 6.71–6.75 (2H, d, J = 12.2 Hz, CH=CH), 6.79–6.81 (2H, d, J = 12.1 Hz, CH=CH), 7.12–7.13 (3H, m ArH), 7.29 (2H, s, ArH), 7.41 (2H, d, J = 6.1 Hz, ArH), 7.43 (2H, d, J = 6.1 Hz, ArH), 9.62 (2H, s, ArOH); ESI-MS: 504.2 (M+1)^+^ 506.1 (M+2)^+^.

#### 4.1.2. 1-(2,6-Dichlorophenyl)-4,6-bis((E)-4-hydroxy-3-methoxystyryl)pyrimidin-2(1H)-one

(**C2**): Mp 148–150 °C; IR (KBr) v_max_: 3402 (OH), 1638 (C=O), 1559 (C=N), 1153 (O-CH_3_), 713 (C-Cl) cm^−^^1^; ^1^H NMR (400 MHz; DMSO-d_6_): δ; 3.81 (6H, s, OCH_3_), 6.04 (1H, s, ArH_pyrimidine_), 6.71–6.75 (2H, d, J = 12.1 Hz, CH=CH), 6.79–6.81 (2H, d, J = 12.0 Hz, CH=CH), 7.12–7.16 (4H, m ArH), 7.30 (2H, s, ArH), 7.50–7.54 (3H, m, ArH), 9.62 (2H, s, ArOH); ESI-MS: 538.2 (M+1)^+^ 540.1 (M+2)^+^.

#### 4.1.3. 1-(3-Chloro-4-fluorophenyl)-4,6-bis((E)-4-hydroxy-3-methoxystyryl)pyrimidin-2(1H)-one

(**C3**): Mp 164–162 °C; IR (KBr) v_max_: 3382 (OH), 1638 (C=O), 1558 (C=N), 1154 (O-CH_3_), 875 (C-Br), 713 (C-Cl) cm^−^^1^; ^1^H NMR (400 MHz; DMSO-d_6_): δ; 3.81 (6H, s, OCH_3_), 6.04 (1H, s, ArH_pyrimidine_), 6.69–6.71 (2H, d, J = 12.0 Hz, CH=CH), 6.77–6.79 (2H, d, J = 12.1 Hz, CH=CH), 6.81–6.99 (4H, m ArH), 7.11 (2H, s, ArH), 7.22 (1H, s, ArH), 7.42–7.44 (2H, m, ArH), 9.62 (2H, s, ArOH); ESI-MS: 522.1 (M+1)^+^ 524.1 (M+2)^+^.

### 4.2. Anticancer Activity

The antiproliferative activity of the curcumin analogues (**C1-C3**) was evaluated against nine diverse panels of 59 cancer cell lines at a single dose (10 µM) and a 5-dose assay (0.001 to 100 µM) according to the National Cancer Institute (NCI US) protocol [43,44,45,46,47,48,49,50]. We explained the detailed method in our previous work [2].

### 4.3. Molecular Docking Studies

The molecular docking against EGFR was performed for the ligands, **C1-C3**. The EGFR (PDB: 2J5F) X-ray crystal structure with a resolution of 3.00 Å; R-value 0.194 (observed) was obtained from the protein data bank (https://www.rcsb.org/structure/2j5f) [55]. The ligands **C1-C3** were saved as mol file and the docking was done as per the protocol reported [52].

## 5. Conclusions

Curcumin was successfully isolated from the ground turmeric powder and chemically modified to prepare three semi-synthetic analogues bearing pyrimidinone nucleus. All the compounds (**C1-C3**) were synthesized in good yields and characterized by analytical data of IR, NMR, and mass spectroscopy. The anticancer activity of curcumin analogues was evaluated in a single-dose experiment at 10 µM. The anticancer activity of compound **C2** was found to be promising with %GI of 68.22 percent, and superior to curcumin, gefitinib, and imatinib against CNS, melanoma, ovarian, prostate, and breast cancer cell lines. The compound **C2** displayed promising anticancer activity and has been further evaluated in 5-dose assay displayed anticancer activity with GI_50_ values ranging from 1.31 to 4.68 µM; however, found to be non-selective with SR values ranging from 0.73 to 1.35. The compound **C2** was found to be more active than curcumin against all nine panels of cancer cell lines. Since EGFR was found to be over-expressed in a number of cancer cell lines, including those from breast, colon, non-small cell lung, renal, melanoma, ovarian, and prostate cancers we selected EGFR as a potential target and the mechanism behind the anticancer activity of the title compounds. We further studied the binding insight of our compounds (**C1-C3**) against the active binding site EGFR where 6-acrylamido-4-anilinoquinazoline usually bind [52]. In molecular docking studies all the ligands were found to accommodate in the active site of EGFR and the binding affinity of ligand **C2** was found to be −5.086 kcal/mol. The ligand **C2** exhibited three different types of interactions: H-bond (Thr790 and Thr854), π-cationic (Arg841), and aromatic H-bond (Asn842). Furthermore, the ligand **C2** displayed good interaction with residues Ala743, Val726, Thr790, Lys745, Leu799, Asp800, Gly721, Leu781, Cys775, Leu844, Cys797, Ser720, and Phe723. The curcumin analogues reported in the current investigation may provide valuable therapeutic intervention for the prevention and treatment of cancer and accelerate anticancer drug discovery programs in the future.

## Figures and Tables

**Table 1:** 60 NCI cancer cell lines based antiproliferative activity of curcumin analogues **C1-C3** in single dose (10 µM) and 5-dose assay (0.001–100 µM) of curcumin analogue **C2**.

Panel	Cell Line	GP and %GI at 10 µM						C2 (NSC 781640)				
		C1 (NSC 781637)		C2 (NSC 781640)		C3 (NSC 799008)		GI_50_	Sub Panel MID ^b^	Selectivity Ratio (MID ^a^: MID ^b^)	TGI	LC_50_
		GP	%GI	GP	%GI	GP	%GI					
***Leukemia***	CCRF-CEM	44.18	**55.82**	10.44	**89.66**	55.57	44.43	3.21	3.06	0.97	70.6	>100
	HL-60(TB)	92.60	7.40	13.53	**86.47**	90.94	9.06	2.64			14.4	>100
	K-562	72.65	17.35	9.21	**90.79**	74.68	25.32	3.11			>100	>100
	MOLT-4	42.97	**57.03**	9.71	**90.29**	52.53	47.47	3.38			22.6	>100
	RPMI-8226	64.47	35.53	0.56	**99.44**	55.35	44.65	2.96			22.7	>100
	SR	36.42	**63.58**	4.12	**95.88**	67.80	32.20	-			-	-
***Non-Small Cell Lung Cancer***	A549/ATCC	98.11	1.89	44.15	**55.85**	78.13	21.87	4.03	3.29	0.91	15.0	52.6
	EKVX	78.11	21.89	41.39	**52.61**	94.55	5.45	3.26			14.8	43.0
	HOP-62	90.11	9.89	50.58	49.42	99.14	0.86	3.41			12.8	37.8
	HOP-92	88.64	11.36	78.69	21.31	-	-	4.22			16.4	83.7
	NCI-H226	91.28	8.72	48.85	**51.15**	102.17	−2.17	3.27			9.44	70.6
	NCI-H23	93.66	6.34	28.96	**71.04**	94.32	5.68	2.38			6.68	59.7
	NCI-H322M	82.00	18.00	72.65	27.35	99.76	0.24	4.14			16.4	47.4
	NCI-H460	92.29	7.71	70.84	29.16	87.46	12.54	3.39			11.8	56.8
	NCI-H522	91.27	8.73	9.89	**90.11**	51.69	48.31	1.55			3.23	67.3
***Colon Cancer***	COLO 205	91.32	8.68	71.81	28.19	114.89	−14.89	2.84	2.19	1.35	8.95	40.5
	HCC-2998	103.85	−3.85	41.77	**58.23**	103.02	−3.02	1.83			3.53	6.82
	HCT-116	50.58	49.42	4.63	**95.37**	61.27	38.73	1.31			5.25	33.2
	HCT-15	85.38	14.62	10.28	**89.72**	92.27	7.73	2.44			9.66	48.4
	HT29	−5.09	**105.09**	−30.44	**130.44**	44.35	55.65	1.77			3.36	6.41
	KM12	76.99	23.01	12.12	**87.88**	86.85	13.15	2.02			5.28	28.7
	SW-620	85.58	14.42	10.43	**89.57**	101.39	−1.39	3.11			1.05	54.1
***CNS Cancer***	SF-268	76.05	23.95	19.85	**80.15**	84.32	15.68	3.12	2.82	1.05	13.7	60.2
	SF-295	97.33	2.67	75.37	25.63	104.21	−4.21	2.87			9.52	32.6
	SF-539	78.08	21.92	3.35	**96.65**	97.11	2.89	2.18			6.92	29.0
	SNB-19	91.04	9.96	34.14	**65.86**	83.03	16.97	3.54			13.2	42.5
	SNB-75	69.72	30.28	49.42	**51.58**	80.29	9.71	-			-	-
	U251	75.53	24.47	8.54	**91.46**	72.05	27.95	2.37			6.36	−22.5
***Melanoma***	LOX IMVI	61.57	38.43	2.83	**97.17**	78.03	21.97	1.68	2.63	1.13	3.57	7.56
	MALME-3M	99.96	0.04	58.79	41.21	91.82	8.18	3.88			15.7	42.2
	M14	88.30	11.70	7.71	42.29	92.06	7.94	2.38			6.87	27.5
	MDA-MB-435	74.93	25.07	4.57	**95.43**	94.71	5.29	1.63			5.98	25.3
	SK-MEL-2	92.56	7.44	82.92	17.08	83.56	16.44	3.30			12.	39.2
	SK-MEL-28	81.58	18.42	17.59	**82.41**	98.97	1.03	2.74			10.1	40.5
	SK-MEL-5	-	-	-	-	83.18	16.82	2.18			4.86	12.3
	UACC-257	96.99	3.01	41.10	**58.9**	67.98	32.02	3.32			11.3	33.7
	UACC-62	80.58	9.42	38.17	**61.83**	75.14	24.86	2.59			7.42	29.3
***Ovarian Cancer***	IGROV1	8.57	**91.43**	33.46	**66.54**	99.85	0.15	3.01	3.32	0.89	12.3	58.5
	OVCAR-3	83.75	16.25	10.66	**89.34**	93.60	6.40	3.19			12.1	>100
	OVCAR-4	78.34	21.66	56.26	43.74	100.98	−0.98	4.61			16.9	41.1
	OVCAR-5	103.44	−3.44	57.58	42.42	114.83	−14.83	2.76			11.0	37.9
	OVCAR-8	107.34	−7.34	7.53	**92.47**	82.79	17.21	3.34			10.5	50.3
	NCI/ADR-RES	96.51	3.49	55.21	44.79	99.97	0.03	3.53			22.0	>100
	SK-OV-3	89.73	9.27	47.71	**52.29**	102.00	−2.00	2.78			10.6	35.1
***Renal Cancer***	786-0	92.56	7.44	29.70	**70.30**	95.36	4.64	2.74	2.93	1.01	8.51	31.0
	A498	102.35	−2.35	78.54	21.46	97.96	2.04	3.79			13.2	36.4
	ACHN	83.10	16.90	37.78	62.22	93.04	6.96	2.84			12.7	66.8
	CAKI-1	92.92	7.08	63.18	36.82	83.20	16.80	1.46			30.0	62.0
	RXF 393	90.70	9.30	11.82	**88.18**	106.20	−6.20	2.18			4.92	>100
	SN 12C	80.80	9.20	27.84	**72.16**	76.04	23.96	4.82			19.5	65.7
	^TK^-10	117.99	−17.99	86.02	13.98	102.66	−2.66	2.91			10.1	36.1
	UO-31	64.88	35.12	25.16	**74.86**	72.46	27.54	2.74			12.3	36.6
***Prostate Cancer***	PC-3	77.28	27.72	34.86	65.14	82.94	17.06	4.68	4.08	0.73	25.3	>100
	DU-145	91.71	8.29	28.74	**71.26**	84.61	16.39	3.48			13.0	37.0
***Breast Cancer***	MCF7	47.62	52.38	13.75	**86.25**	67.34	32.66	2.00	3.23	0.92	11.0	78.2
	MDA-MB-231	60.31	39.69	29.00	**71.00**	65.84	34.16	3.64			15.1	>100
	HS 578T	87.44	12.66	36.31	**63.69**	85.47	14.53	5.48			>100	>100
	BT-549	66.90	33.10	15.17	**84.83**	80.77	9.23	2.07			66.7	28.6
	T-47D	85.96	14.04	24.11	**75.89**	82.52	7.48	3.44			22.7	>100
	MDA-MB-468	76.44	23.56	29.59	**70.41**	89.61	10.39	2.76			7.19	44.5
Mean		79.63	20.37	31.78	**68.22**	85.67	14.33	2.97				
Range (% GI)		−17.99 to 105.09		13.98 to 130.44		−14.83 to 55.65						
Total number of cell lines and sum of concentration (µM)	59							58/172.3				
MID ^a^								2.971				

% GI = 100—GP; (-) = Not tested; Black font = < 50% GI; Bold & blue font = GIs > 50 (GPs < 50); Bold & green font = GIs > 68 (GPs < 32); Bold & red font = GIs >100 (GPs = < 0 means lethal effect) (One dose assay); NSC Number: Originally known as Cancer Chemotherapy National Service Center number, and it is an identifying number assigned by Developmental therapeutics Program (DTP) to an agent or product (e.g., small molecule or biological agent); MID ^a^ and MID ^b^ were the mean GI_50_ of 60 NCI cancer cell lines and the individual cancer cell line panels; Selectivity ration = MID ^a^/MID ^b^; GI_50_, LC_50_ and TGI stand for 50% growth inhibition, 50% lethal concentration, and total growth inhibition respectively.

**Table 2:** Mean % GI of compounds **C1-C3** and curcumin at 10 µM.

Panel	C1	C2	C3	Cur *	Gefitinib ^#^	Imatinib ^#^
*Leukemia*	39.45	92.09	33.86	**97.76**	79.68	9
*Non-Small cell lung cancer*	10.50	49.78	11.60	49.27	**63.97**	15.68
*Colon Cancer*	30.20	82.77	13.71	**95.76**	52.19	5.34
*CNS Cancer*	18.88	**68.56**	11.50	60.75	46.13	5.8
*Melanoma*	14.19	**62.04**	14.95	54.63	44.99	−0.87
*Ovarian Cancer*	18.76	**61.66**	0.85	44.66	60.93	−7.16
*Renal Cancer*	8.09	54.99	9.14	45.35	**77.89**	3.25
*Prostate Cancer*	18.01	**68.20**	16.73	61.3	59.6	12.5
*Breast Cancer*	29.24	**75.35**	18.08	56.1	52.88	12.15

* Cur = Curcumin; the anticancer activity of curcumin was retrieved from NCI data-ware with NSC ID 32,982 [46]; bold font represents the best result; ^#^ The anticancer activity of gefitinib and imatinib were retrieved from NCI data-ware with NSC ID 759,856 and 759,854 respectively [46].

**Table 3:** The molecular docking results of curcumin analogues against EGFR.

S. No.	Compound	2D Docking	Docking Score	Glide Emodel	Interaction
1	**C1**	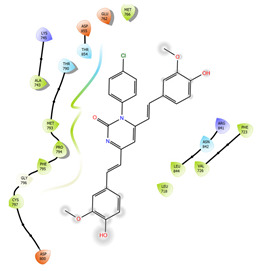	−5.117	−66.925	Aromatic H-Bond (Asp855)
2	**C2**	** 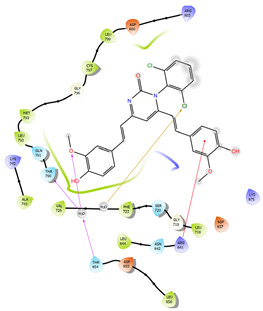 **	−5.086	−62.292	H-Bond (Thr790 and Thr854); π-Cationic (Arg841); Aromatic H-Bond (Asn842)
3	**C3**	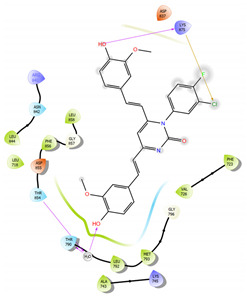	−4.396	−66.538	H-Bond (Thr790, Thr854, Lys875); Halogen Bond (Lys875); Aromatic H-Bond (Asp855)

## Data Availability

The authors confirm that the data supporting the study’s findings are included in the article and its supplementary information.

## Supplementary Materials

The following supporting information can be downloaded at: https://www.mdpi.com/article/10.3390/plants11202737/s1, Figure S1–S3: Anticancer data of compounds C1-C3 against 59 cancer cell lines., Figure S4. 3D Interaction of ligand C1 against the binding site EGFR.
