# Supplementary material for: Chemical Modification of Curcumin into Its Semi-Synthetic Analogs Bearing Pyrimidinone Moiety as Anticancer Agents
Source: Plants (Basel). 2022 Oct 16;11(20):2737. doi: 10.3390/plants11202737 (PMC9607420; doi:10.3390/plants11202737)
Supplement: Supplementary file 1 [file plants-11-02737-s001.zip › plants-1952218-supplementary.pdf]

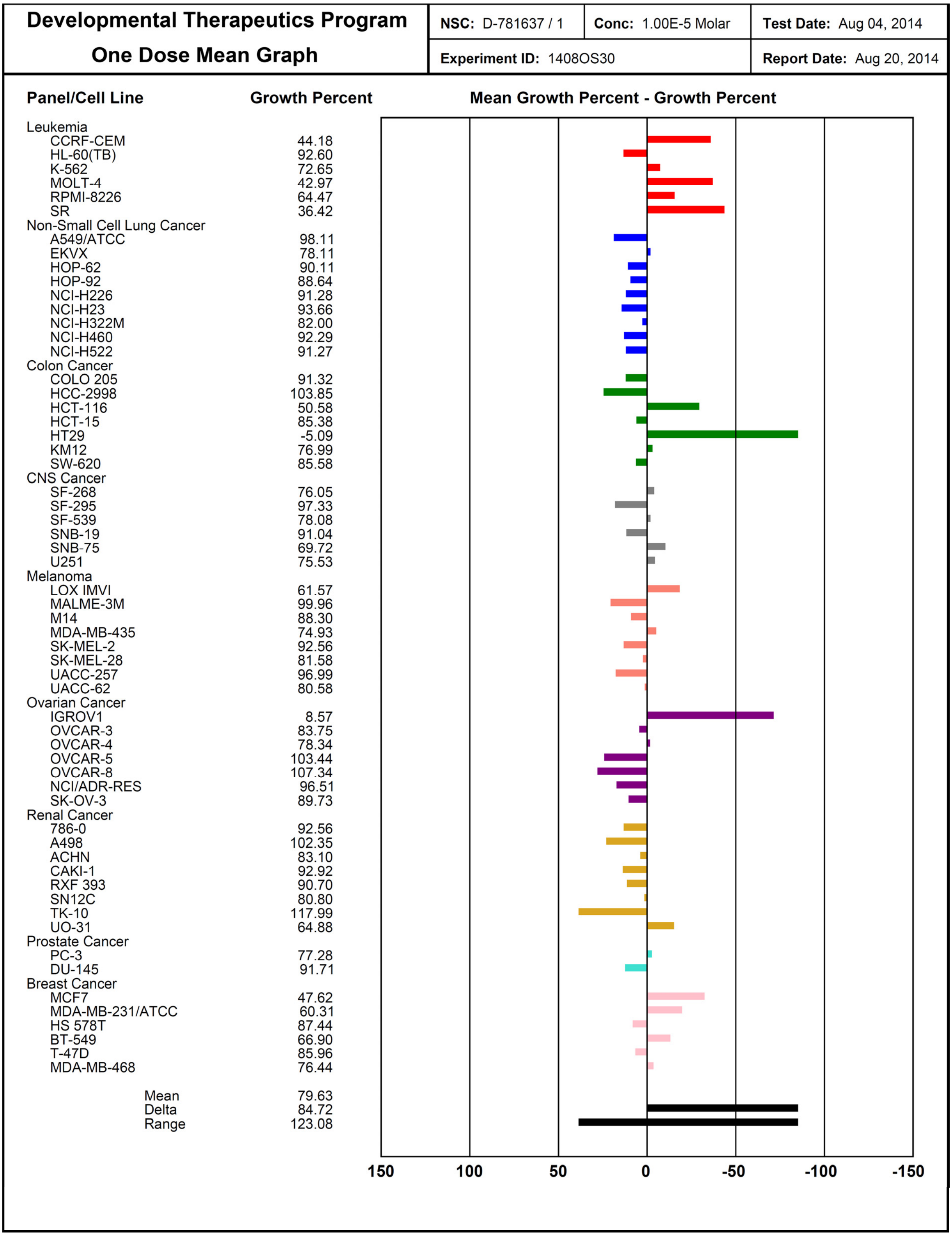

Figure S1. Anticancer data of compound **C1** against 59 cancer cell lines.

Developmental Therapeutics Program
One Dose Mean Graph

**NSC:** D-781640 / 1	**Conc:** 1.00E-5 Molar
**Test Date:** Aug 04, 2014	**Experiment ID:** 1408OS30
**Report Date:** Aug 20, 2014	

Panel/Cell Line	Growth Percent
Leukemia	
CCRF-CEM	10.44
HL-60(TB)	13.53
K-562	9.21
MOLT-4	9.71
RPMI-8226	0.56
SR	4.12
Non-Small Cell Lung Cancer	
A549/ATCC	44.15
EKVX	41.39
HOP-62	50.58
HOP-92	78.69
NCI-H226	48.85
NCI-H23	28.96
NCI-H322M	72.65
NCI-H460	70.84
NCI-H522	9.89
Colon Cancer	
COLO 205	71.81
HCC-2998	41.77
HCT-116	4.63
HCT-15	10.28
HT29	-30.44
KM12	12.12
SW-620	10.43
CNS Cancer	
SF-268	19.85
SF-295	75.37
SF-539	3.35
SNB-19	34.14
SNB-75	49.42
U251	8.54
Melanoma	
LOX IMVI	2.83
MALME-3M	58.79
M14	7.71
MDA-MB-435	4.57
SK-MEL-2	82.92
SK-MEL-28	17.59
UACC-257	41.10
UACC-62	38.17
Ovarian Cancer	
IGROV1	33.46
OVCAR-3	10.66
OVCAR-4	56.26
OVCAR-5	57.58
OVCAR-8	7.53
NCI/ADR-RES	55.21
SK-OV-3	47.71
Renal Cancer	
786-0	29.70
A498	78.54
ACHN	37.78
CAKI-1	63.18
RXF 393	-11.82
SN12C	27.84
TK-10	86.02
UO-31	25.16
Prostate Cancer	
PC-3	34.86
DU-145	28.74
Breast Cancer	
MCF7	13.75
MDA-MB-231/ATCC	29.00
HS 578T	36.31
BT-549	15.17
T-47D	24.11
MDA-MB-468	29.59
Mean	31.78
Delta	62.22
Range	116.46

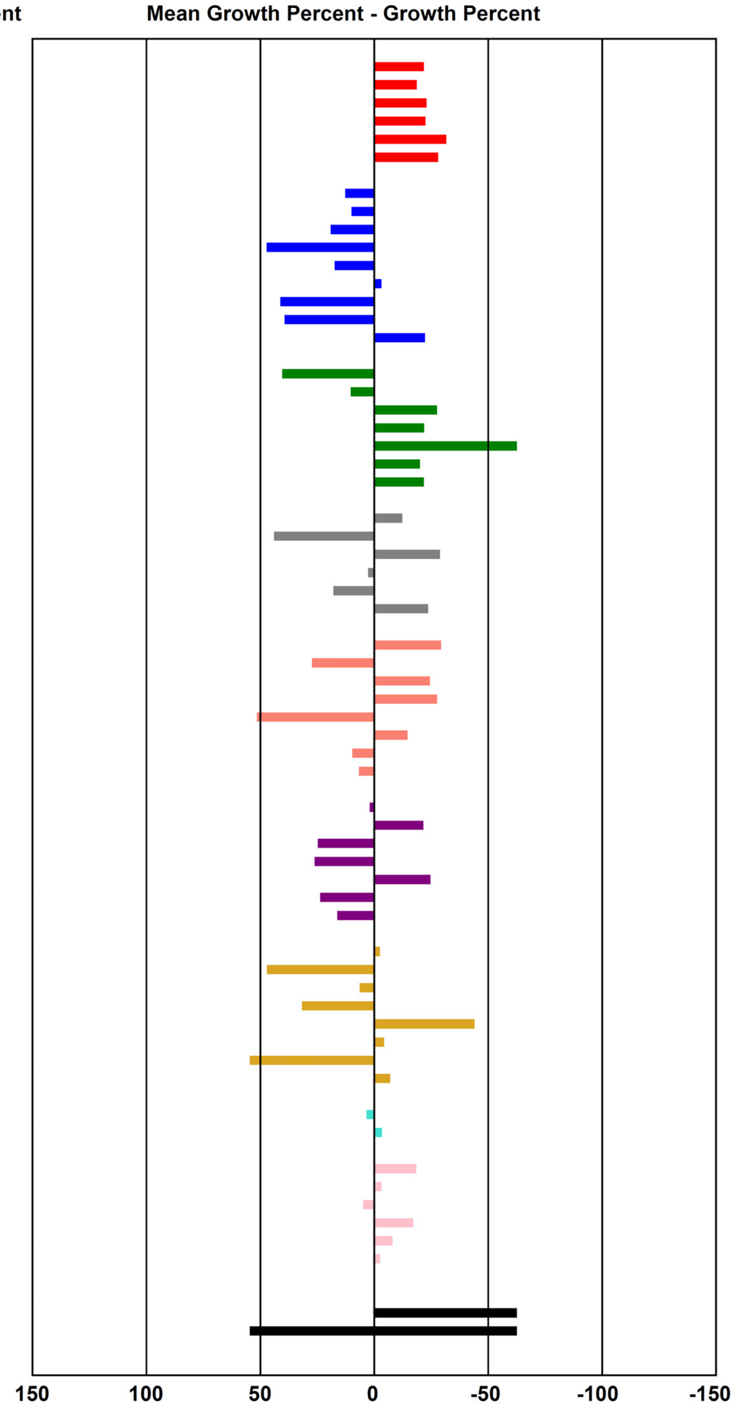

Figure S2. Anticancer data of compound **C2** against 59 cancer cell lines.

Developmental Therapeutics Program One Dose Mean Graph	NSC: D-799008 / 1	Conc: 1.00E-5 Molar	Test Date: Jul 10, 2017
	Experiment ID: 1707OS32		Report Date: Aug 03, 2017

Panel/Cell Line	Growth Percent
**Leukemia**	
CCRF-CEM	55.57
HL-60(TB)	90.94
K-562	74.68
MOLT-4	52.53
RPMI-8226	55.35
SR	67.80
**Non-Small Cell Lung Cancer**	
A549/ATCC	78.13
EKVX	94.55
HOP-62	99.14
NCI-H226	102.17
NCI-H23	94.32
NCI-H322M	99.76
NCI-H460	87.46
NCI-H522	51.69
**Colon Cancer**	
COLO 205	114.89
HCC-2998	103.02
HCT-116	61.27
HCT-15	92.43
HT29	44.35
KM12	86.85
SW-620	101.39
**CNS Cancer**	
SF-268	84.32
SF-295	104.21
SF-539	97.11
SNB-19	83.03
SNB-75	80.29
U251	72.05
**Melanoma**	
LOX IMVI	78.03
MALME-3M	91.82
M14	92.06
MDA-MB-435	94.71
SK-MEL-2	83.56
SK-MEL-28	98.97
SK-MEL-5	83.18
UACC-257	67.98
UACC-62	75.14
**Ovarian Cancer**	
IGROV1	99.85
OVCAR-3	93.60
OVCAR-4	100.98
OVCAR-5	114.83
OVCAR-8	82.79
NCI/ADR-RES	99.97
SK-OV-3	102.00
**Renal Cancer**	
786-0	95.36
A498	97.96
ACHN	93.04
CAKI-1	83.20
RXF 393	106.20
SN12C	76.04
TK-10	102.66
UO-31	72.46
**Prostate Cancer**	
PC-3	82.94
DU-145	84.61
**Breast Cancer**	
MCF7	67.34
MDA-MB-231/ATCC	65.84
HS 578T	85.47
BT-549	80.77
T-47D	82.52
MDA-MB-468	89.61
Mean	85.67
Delta	41.32
Range	70.54

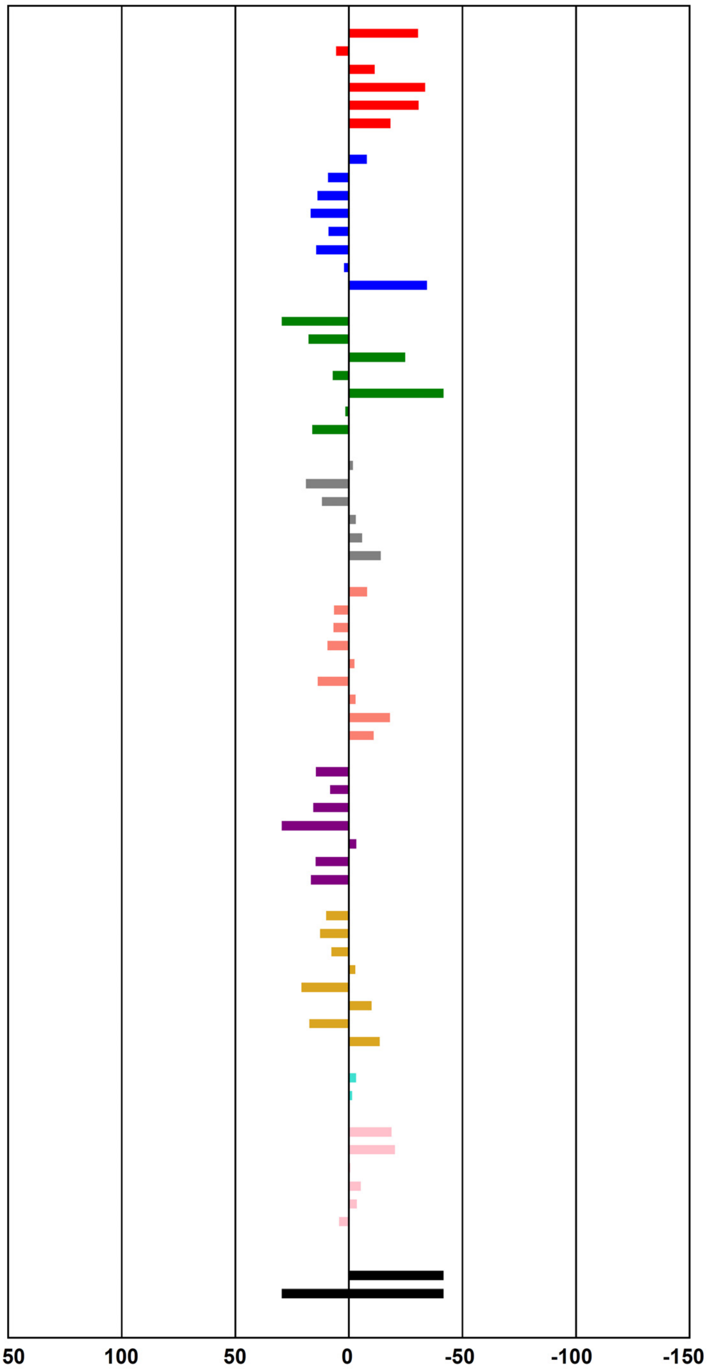

Figure S3. Anticancer data of compound **C3** against 59 cancer cell lines.

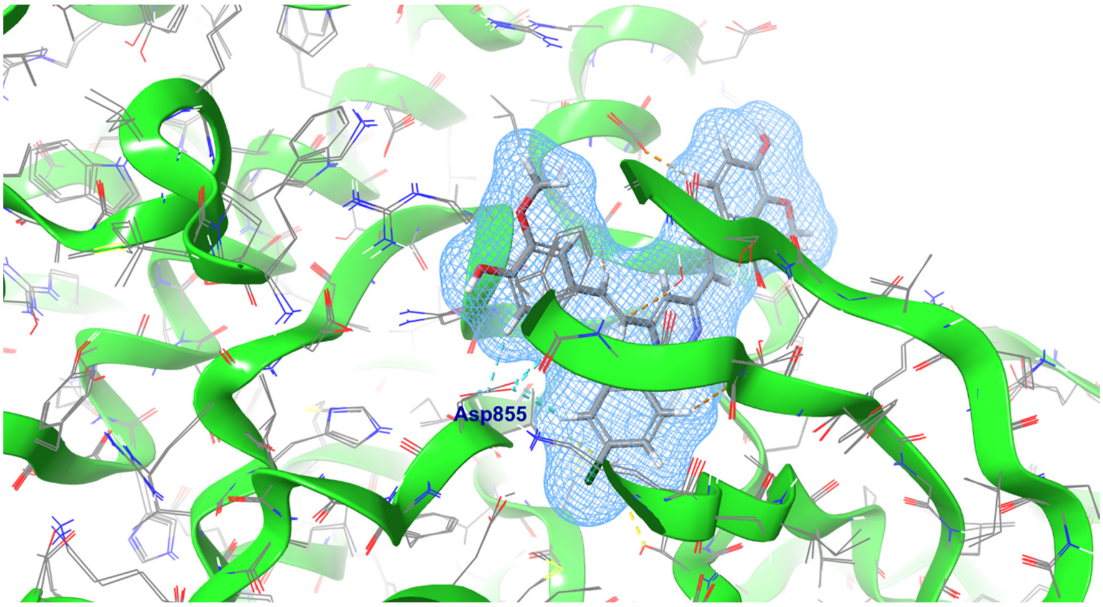

Figure S4. 3D Interaction of ligand **C1** against the binding site EGFR (binding affinity = −5.117 kcal/mol).
